# Idiopathic Renal Infarct in a Healthy Adult With an Accessory Renal Artery: A Case Report

**DOI:** 10.7759/cureus.102806

**Published:** 2026-02-01

**Authors:** Ahmad Haj Hussein, Charbel Ghosn, Richard Feghaly, Mariana Helou

**Affiliations:** 1 Emergency Department, Lebanese American University School of Medicine, Beirut, LBN

**Keywords:** emergency, idiopathic renal infarct, kidney, kidney disease, renal cortical infarct

## Abstract

Renal infarction is an uncommon and frequently underdiagnosed condition caused by the acute interruption of renal arterial blood flow. Its clinical presentation often mimics renal colic, and laboratory findings may be nonspecific, leading to delayed or missed diagnosis. Although embolic and thrombotic etiologies are most commonly implicated, idiopathic renal infarction remains rare.

We report the case of a 45-year-old previously healthy man who presented with sudden-onset left flank pain radiating to the left upper quadrant, associated with nausea and abdominal discomfort. He had no history of cardiovascular disease, trauma, thrombophilia, or autoimmune disorders. Physical examination revealed left costovertebral angle tenderness. Laboratory evaluation demonstrated mild leukocytosis and microscopic hematuria without inflammatory marker elevation. Contrast-enhanced computed tomography of the abdomen and pelvis revealed multifocal wedge-shaped hypoenhancing areas in the left kidney consistent with renal infarction, secondary to filling defects in the main left renal artery. An accessory left renal artery supplying the inferior pole was noted. Autoimmune and thrombophilia workup was negative. The patient was treated with anticoagulation using unfractionated heparin followed by apixaban. Follow-up imaging demonstrated partial recanalization of the renal artery with no progression of infarction and preserved renal function.

Idiopathic renal infarction should be considered in patients presenting with acute flank pain when initial evaluation for nephrolithiasis is negative. Early contrast-enhanced imaging is crucial for diagnosis. Prompt anticoagulation can prevent progression and preserve renal function.

## Introduction

Renal infarct is an uncommon serious clinical entity resulting from an abrupt interruption of blood flow in the branches of the renal arteries, leading to ischemic necrosis of the renal parenchyma [1,2]. Its reported incidence is low; however, this is likely an underestimation due to frequent misdiagnosis. Retrospective studies of emergency admissions reported an incidence of 0.004-0.007% [3]. Therefore, autopsy studies may be more accurate, with an incidence found of 1.4% [3]. The most common cause of renal infarction is thromboembolic events originating from the heart, particularly in patients with atrial fibrillation, valvular heart disease, or recent myocardial infarction, as well as embolization from the proximal aorta. This accounts for up to 55% of cases of renal infarct. Other causes include in situ thrombosis due to atherosclerosis, fibromuscular dysplasia, renal artery dissection, vasculitis, hypercoagulable states, and traumatic or iatrogenic injury [2]. Clinically, renal infarct presents with sudden-onset flank or abdominal pain, frequently associated with nausea, vomiting, fever, or hematuria [3]. Laboratory findings are often nonspecific. Diagnosis is often missed due to normal laboratory findings [4]. Frank or microscopic hematuria can be found in almost all patients [5]. Imaging plays a pivotal role in diagnosis. Contrast-enhanced computed tomography (CT) remains the diagnostic modality of choice, typically demonstrating wedge-shaped, hypoenhancing cortical defects corresponding to areas of infarction [6]. Management of renal infarction depends on the underlying etiology, timing of diagnosis, and extent of renal involvement. Anticoagulation remains the cornerstone of treatment in thromboembolic and idiopathic cases, while endovascular or surgical revascularization may be considered in select patients presenting early with complete arterial occlusion. A delayed or missed diagnosis can result in an irreversible loss of kidney function. Prognosis is improved with early diagnosis and management [3]. We report a case of a 45-year-old man presenting with an idiopathic renal infarct in the absence of thrombophilia or autoimmune disease.

## Case presentation

This is the case of a 45-year-old man previously healthy, presenting with acute-onset left flank pain, one hour prior to presentation, radiating to the left upper quadrant. He reports nausea, abdominal bloating, and discomfort, but denies any fever, chills, hematuria, dysuria, urinary frequency, previous episodes of renal colic, or any other respiratory, intestinal, or urinary symptoms.

The patient has no allergies, no past medical history, no past surgical history, and no family history of hematological diseases or cancers. He denied any substance abuse or intravenous injections.

Vital signs upon presentation showed the following: blood pressure 161/104 mmHg, heart rate 74 bpm, temperature 37.1°C, oxygen saturation 98% on room air, and hemoglucotest 110. Body mass index is 29.65 kg/m². On physical exam, the patient was conscious, cooperative, and oriented. Lung exam showed good bilateral airway entry, with no added sounds on auscultation. Heart sounds were regular with no murmurs. The abdomen is soft, non-tender, and non-distended. He had left costovertebral angle tenderness. Peripheral pulses were 2+, and there was no lower limb edema.

The patient was in significant pain and discomfort. Paracetamol, scopolamine, and nefopam provided minimal relief. Dexketoprofen was given and provided moderate relief. Laboratory tests done upon presentation are shown in detail in Table 1. 

**Table 1:** Laboratory data upon admission

Test	Result	Reference range
Corrected white blood cell (WBC)	11.09×10³/µL	5.2-12.4×10³/µL
Hemoglobin (HGB)	15.3 g/dL	13.6-17.5 g/dL
Hematocrit (HCT)	44.3%	41-53%
Platelets (PLT)	214×10³/µL	150-400×10³/µL
Sodium (S)	143 mmol/L	136-145 mmol/L
Potassium (S)	3.8 mmol/L	3.5-5.1 mmol/L
Chloride (S)	104 mmol/L	98-107 mmol/L
CO₂ (S)	25 mmol/L	22-29 mmol/L
Blood urea nitrogen (BUN) (S)	17.2 mg/dL	6-20 mg/dL
Creatinine (S)	1.14 mg/dL	0.7-1.2 mg/dL
Estimated glomerular filtration rate (/1.73 m²)	81 mL/min/1.73 m²	>60 mL/min/1.73 m²
Gamma-glutamyl transferase (S)	143 U/L	10-71 U/L
Alkaline phosphatase (S)	104 U/L	40-130 U/L
Alanine aminotransferase (S)	78 U/L	<42 U/L
Aspartate aminotransferase (S)	35 U/L	<41 U/L
Creatine phosphokinase (CPK) (S)	130 U/L	<190 U/L
Lipase (S)	27 U/L	13-60 U/L
Bilirubin direct (S)	0.4 mg/dL	≤0.2 mg/dL
Bilirubin indirect (S)	0.7 mg/dL	0.2-1 mg/dL
Bilirubin total (S)	1.1 mg/dL	0.2-1 mg/dL
C-reactive protein (CRP)	1.3 mg/L	<5 mg/L
Prothrombin time	15.3 sec	-
International normalized ratio (INR)	1.14	<1.2
Activated partial thromboplastin time (aPTT)	26 sec	<36 sec
D-dimer	0.27 µg/mL	<0.5 µg/mL
Urinalysis		
Red blood cells (RBCs)	3-4/HPF	0-2/HPF
White blood cells (WBCs)	1-3/HPF	0-5/HPF

**Figure 1:**
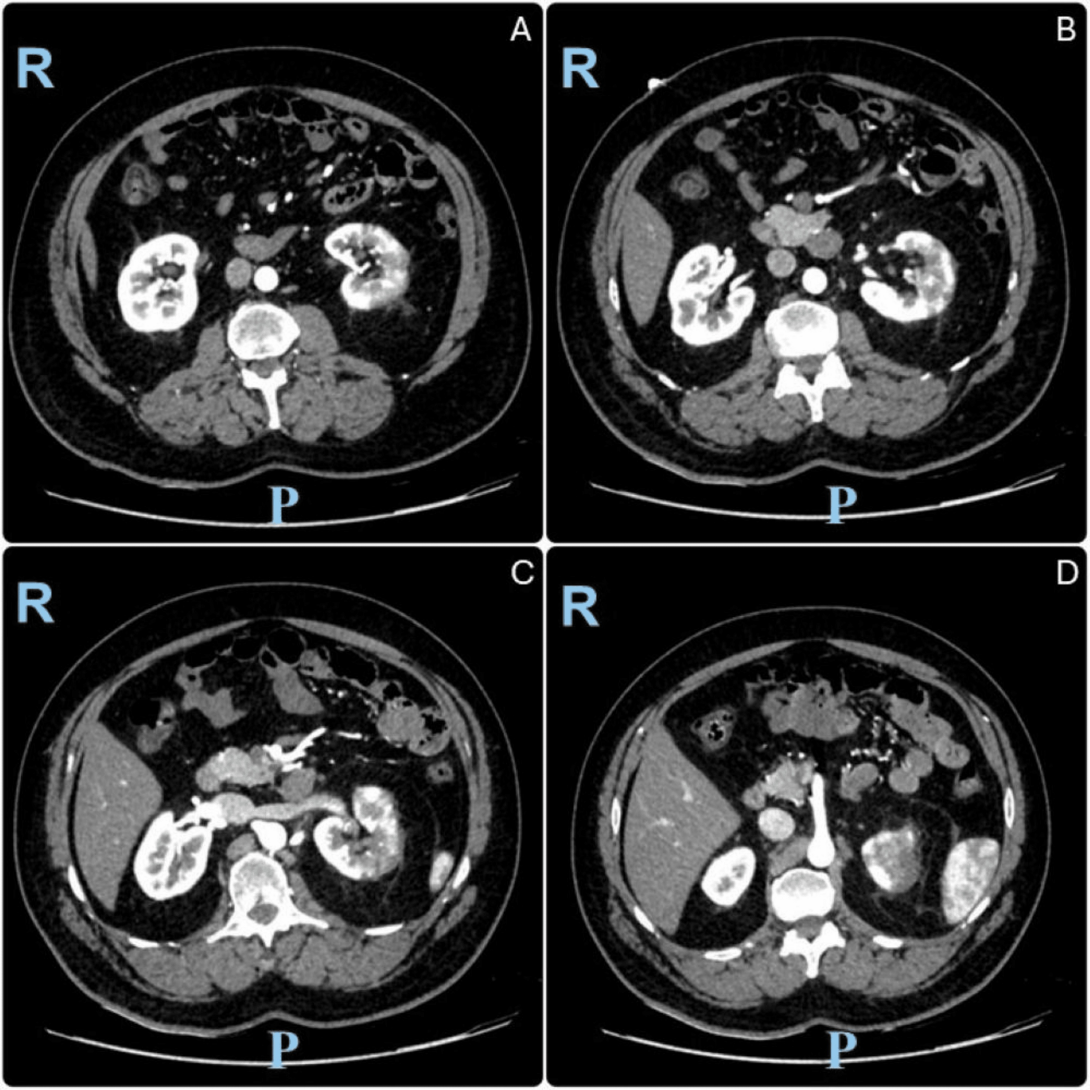
Axial view computed tomography of the abdomen and pelvis with intravenous contrast in the arterial phase showing multifocal left renal infarcts Images are ordered in a caudocephalic direction. R: right side; P: posterior side

**Figure 2:**
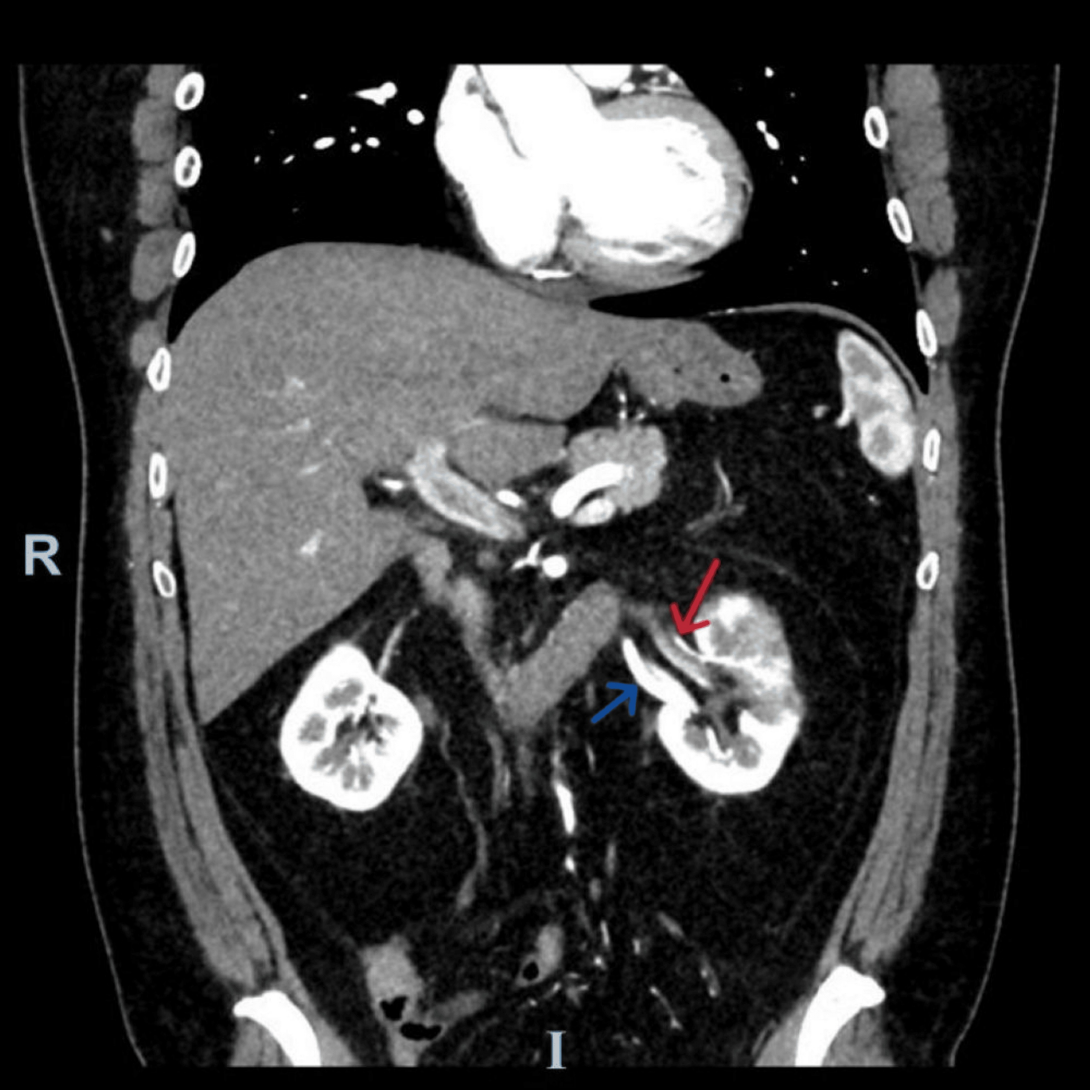
Coronal view computed tomography of the abdomen and pelvis with intravenous contrast in the arterial phase showing the two left renal arteries Red arrow: The main renal artery supplies the upper and mid poles and demonstrates multiple filling defects within its lumen. Blue arrow: The accessory renal artery supplies the lower pole and appears to be patent.

A CT scan of the abdomen and pelvis with intravenous contrast was done. The scan showed multifocal wedge-shaped hypoenhancing areas within the left kidney, predominantly at the upper pole. There are two left-sided renal arteries. The main renal artery supplies the upper and mid poles and demonstrates multiple filling defects within its lumen. The accessory renal artery supplies the lower pole and appears to be patent. These imaging ruled out the possibility of isolated renal artery dissection as an absence of a false lumen or intimal flap. Overall findings are suggestive of renal infarcts. Different axial cuts are shown in Figure 1, while Figure 2 clearly shows the left accessory renal artery.

The patient was then admitted to the medical-surgical unit (MSU) and was started on unfractionated heparin adjusted to his activated partial thromboplastin time (aPTT). Autoimmune and thrombophilia workup was negative, including negative p-ANCA (anti-myeloperoxidase) and c-ANCA (anti-PR3), negative ANA by immunofluorescence, and negative anti-β2 glycoprotein I antibodies. Molecular testing showed no evidence of factor V Leiden mutation, ruling out inherited activated protein C resistance.

**Figure 3:**
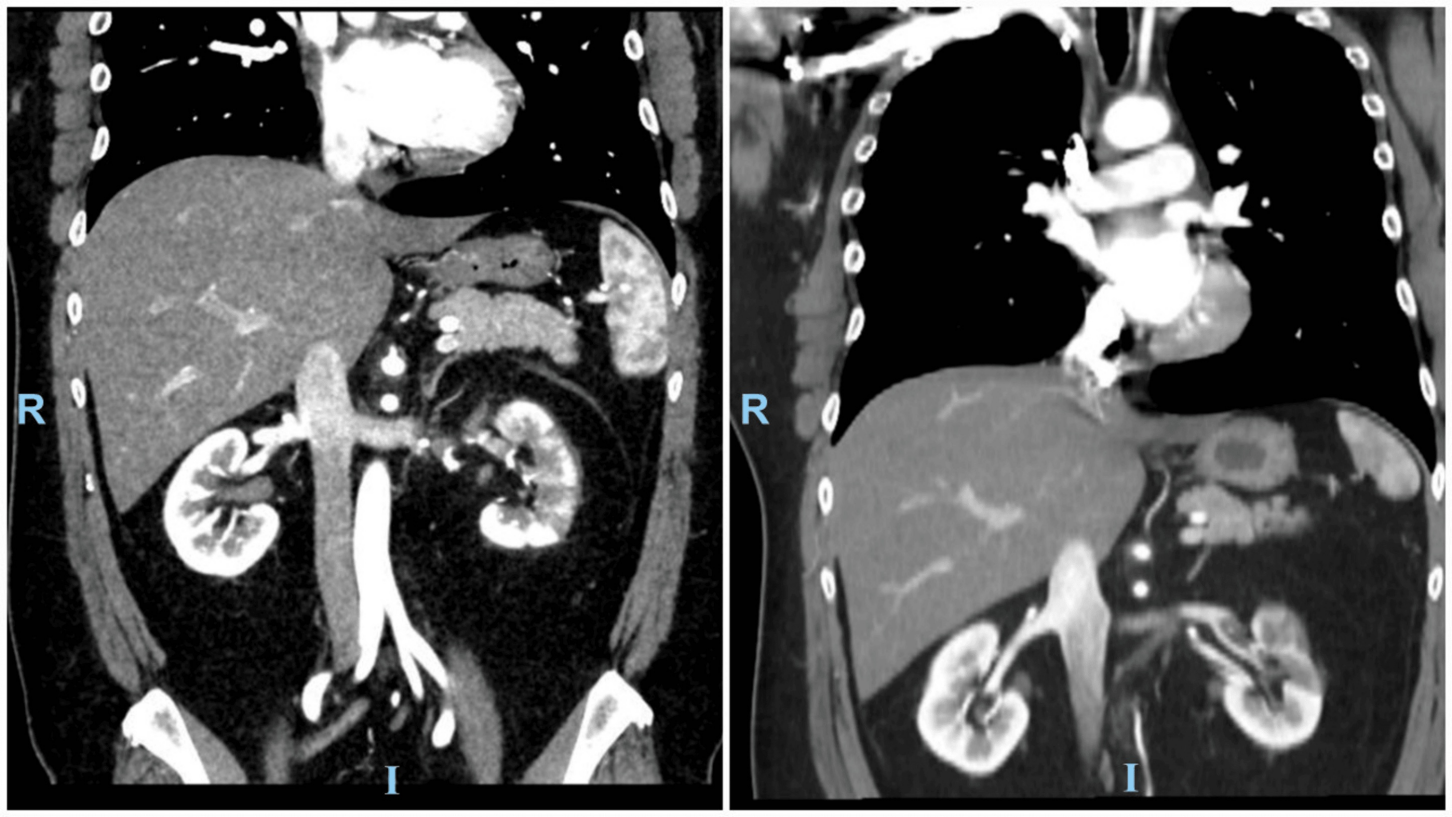
Follow-up computed tomography angiography of the aortoiliac after three weeks showing persistent filling defect with better recanalization of the left renal artery Left image: computed tomography scan upon presentation. Right image: computed tomography scan after three weeks

A follow-up CT angiography of the aortoiliac showed a persistent filling defect within the mid and distal portion of the left renal artery, resulting in subsequent incomplete occlusion (Figure 3). There was also an interval increase in the size of multifocal left-sided renal parenchymal infarcts. The apical segmental branch of the left renal artery appears patent, as does a left accessory renal artery supplying the inferior pole.** **Follow-up labs showed a mild increase in levels of creatinine from 1.14 mg/dL to 1.32 mg/dL. The patient was discharged against medical advice on day 2 of admission on Eliquis 5 mg BID.

After three weeks, the patient returned for a follow-up CT scan that showed a persistent filling defect with better recanalization of the left renal artery, resulting in subsequent incomplete occlusion. Multifocal left-sided renal parenchymal infarcts were again noted.

## Discussion

This case describes a middle-aged man with no identifiable risk factors or family history and no abnormalities on cardiac or peripheral arterial ultrasound examinations, who presented with symptoms mimicking renal colic in the absence of hematuria or other urinary symptoms and was ultimately found to have multiple renal infarcts. Immunological and autoimmune markers, antinuclear antibodies, and rheumatoid factor were normal. Aside from a history of smoking, the etiology of the infarct was undetermined, leading to a diagnosis of idiopathic renal infarction, a very rare condition. In searching for a cause, a systematic review linked SARS-CoV-2-induced thrombosis to renal infarction [7]. Other case reports found an embolizing traumatic post-dissection aneurysm of a renal segmental artery 1.5 years after a motorcycle accident or sickle cell disease as causes of renal infarction [8,9]. However, our case had no respiratory infection nor a high-energy trauma or sickle cell disease.

Clinical presentation is often variable, but almost always includes acute-onset flank pain and hematuria, with some cases reporting abdominal pain and nausea [3]. In the case presented above, the patient reported acute-onset flank pain with nausea and vague abdominal distension. However, he had no hematuria and neither frank blood nor microscopic hematuria. Perhaps this is due to his fast response in seeking treatment post-flank pain and a small delay prior to diagnosis.

The presence of an accessory renal artery was an incidental finding as the patient did not have a prior CT scan. This artery represents a distinct segmental supply to the renal parenchyma, consistent with normal variations in renal vascular anatomical territory. Patients with acute kidney infarct usually suffer from an acute kidney injury (AKI) that transforms into chronic kidney disease (CKD). Our patient's mild creatinine elevation was probably due to the remaining blood flow from the accessory artery.

Treatment is often through anticoagulation and hemodialysis. Surgery is sometimes needed to remove the clot, but is effective only in the first hours [6]. Our patient was started on anticoagulation (unfractionated heparin) and was discharged on direct oral anticoagulants (DOACs) (Eliquis 5 mg BID). This therapy was effective and evident by the decrease in infarct size on follow-up CT scan three weeks after his presentation.

The long-term outcomes of renal infarction depend on the severity and extent of the renal infarction. CKD and reinfarction are the most frequent documented complications seen after long-term follow-up [1,10]. After a four-week follow-up, the patient is still on anticoagulation. He had no episodes of reinfarction. CT showed the resolution of the old clot. Creatinine levels returned back to normal after a small rise and AKI.

## Conclusions

Idiopathic renal infarct is an uncommon condition. It should be considered in patients presenting with acute flank pain when initial evaluation for nephrolithiasis is negative. Early contrast-enhanced imaging is crucial for diagnosis. Prompt anticoagulation can prevent progression and preserve renal function. Treatment aims to remove the clot, either surgically or medically through anticoagulation. Long-term outcomes depend on the severity and extent of renal infarction and the collateral blood supply. The accessory renal artery, which was once treated as an anomaly, was able to save this patient from dialysis.
